# P2 Receptors as Therapeutic Targets in the Salivary Gland: From Physiology to Dysfunction

**DOI:** 10.3389/fphar.2020.00222

**Published:** 2020-03-13

**Authors:** Mahmoud G. Khalafalla, Lucas T. Woods, Kimberly J. Jasmer, Kevin Muñoz Forti, Jean M. Camden, Janicke L. Jensen, Kirsten H. Limesand, Hilde K. Galtung, Gary A. Weisman

**Affiliations:** ^1^Department of Biochemistry, University of Missouri, Columbia, MO, United States; ^2^Christopher S. Bond Life Sciences Center, University of Missouri, Columbia, MO, United States; ^3^Department of Medicine, Feinberg School of Medicine, Northwestern University, Chicago, IL, United States; ^4^Institute of Clinical Dentistry, Section of Oral Surgery and Oral Medicine, University of Oslo, Oslo, Norway; ^5^Department of Nutritional Sciences, University of Arizona, Tucson, AZ, United States; ^6^Institute of Oral Biology, Faculty of Dentistry, University of Oslo, Oslo, Norway

**Keywords:** purinergic receptors, saliva, salivary gland dysfunction, Sjögren’s syndrome, extracellular nucleotides, head and neck cancer

## Abstract

Although often overlooked in our daily lives, saliva performs a host of necessary physiological functions, including lubricating and protecting the oral cavity, facilitating taste sensation and digestion and maintaining tooth enamel. Therefore, salivary gland dysfunction and hyposalivation, often resulting from pathogenesis of the autoimmune disease Sjögren’s syndrome or from radiotherapy of the head and neck region during cancer treatment, severely reduce the quality of life of afflicted patients and can lead to dental caries, periodontitis, digestive disorders, loss of taste and difficulty speaking. Since their initial discovery in the 1970s, P2 purinergic receptors for extracellular nucleotides, including ATP-gated ion channel P2X and G protein-coupled P2Y receptors, have been shown to mediate physiological processes in numerous tissues, including the salivary glands where P2 receptors represent a link between canonical and non-canonical saliva secretion. Additionally, extracellular nucleotides released during periods of cellular stress and inflammation act as a tissue alarmin to coordinate immunological and tissue repair responses through P2 receptor activation. Accordingly, P2 receptors have gained widespread clinical interest with agonists and antagonists either currently undergoing clinical trials or already approved for human use. Here, we review the contributions of P2 receptors to salivary gland function and describe their role in salivary gland dysfunction. We further consider their potential as therapeutic targets to promote physiological saliva flow, prevent salivary gland inflammation and enhance tissue regeneration.

## Introduction

Salivary gland dysfunction and the associated hyposalivation are serious clinical problems that impact millions of people (Atkinson et al., 2005; Qin et al., 2015; Siddiqui and Movsas, 2017). Saliva plays a crucial role in maintaining oral homeostasis by aiding in taste perception and digestion, protecting and lubricating oral tissues, maintaining the integrity of tooth enamel and sustaining the oral microbiome (Dawes et al., 2015). In addition to its physiological roles, saliva contains a plethora of biomarkers and is easy to access allowing clinicians to utilize saliva as a non-invasive diagnostic material to monitor patient health (Chojnowska et al., 2018). Human saliva is increasingly being used to perform screening and risk assessment for systemic diseases, such as HIV, cancer, infections and cardiovascular disorders, demonstrating saliva’s extensive clinical potential (Nunes et al., 2015). Adequate saliva production is essential for maintaining quality of life and salivary gland dysfunction leads to dry mouth, oral bacterial and yeast infections, dental caries and speech problems (Chambers et al., 2004; Meijer et al., 2009).

Hyposalivation and xerostomia (i.e., dry mouth) can present in an iatrogenic manner as side effects of over 400 medications, including antidepressants, antipsychotics, opioids, antihistamines, and others (Furness et al., 2011). Although often transient and reversible, iatrogenic xerostomia contributes to patient non-adherence to medication regimens leaving underlying pathologies untreated. Two common pathophysiological causes of salivary gland dysfunction in humans are Sjögren’s syndrome (SS), an autoimmune disease characterized by xerostomia, autoantibody production and chronic lymphocytic infiltration of the salivary glands (i.e., sialadenitis), and radiotherapy-induced dysfunction where salivary glands sustain collateral damage following γ-radiation to treat head and neck tumors (Pinna et al., 2015; Mariette and Criswell, 2018). In both cases, damage to the salivary parenchyma and the failure to repair saliva-producing salivary acinar epithelium contribute to glandular dysfunction. Current therapies for salivary gland dysfunction are primarily focused on symptom management using muscarinic receptor agonists (i.e., pilocarpine or cevimeline) to stimulate saliva flow from residual salivary epithelium or through the topical use of artificial saliva (Ramos-Casals et al., 2010). While these treatments can provide some relief to patients, they are relatively ineffective because of their transient nature and failure to address the underlying inflammatory and degenerative processes that initiate and sustain glandular tissue damage. Therefore, a better understanding of the pathophysiology of salivary gland dysfunction is crucial to developing novel therapeutic approaches for this serious medical problem.

Purinergic receptors for extracellular nucleosides (i.e., adenosine) or nucleotides (i.e., ATP, ADP, UTP, UDP, and UDP-glucose) mediate numerous physiological processes, including platelet aggregation, neurotransmission, bone remodeling, and inflammatory, and immune responses (Dorsam and Kunapuli, 2004; Orriss et al., 2010; Idzko et al., 2014; Mutafova-Yambolieva and Durnin, 2014; Verkhratsky and Burnstock, 2014). In exocrine tissues, such as salivary gland, lacrimal gland and pancreas, purinergic receptor-mediated ion fluxes and cross-talk with muscarinic receptor signaling have been suggested to modulate secretory function (Novak et al., 2010; Burnstock and Novak, 2012; Hodges and Dartt, 2016). Whereas intracellular nucleotides are well-known for their role in metabolism and enzyme function, it wasn’t until the 1970s that plasma membrane receptors were postulated to respond to extracellular nucleotides, including ATP and ADP, and were suggested to be responsible for non-cholinergic, non-adrenergic neurotransmission (Burnstock et al., 1972; Burnstock, 1976). Under normal conditions, extracellular nucleotides are present at minute concentrations due to the presence of ectonucleotidases (Robson et al., 2006; Zimmermann et al., 2012). However, under pathological conditions nucleotides can accumulate in the extracellular space at abnormally high concentrations, whereupon they activate local purinergic receptors in an autocrine or paracrine manner (Deaglio and Robson, 2011). The purinergic receptor family is subclassified into P1 adenosine receptors (i.e., A_1_, A_2A_, A_2B_, and A_3_) (Piirainen et al., 2011) or P2 nucleotide receptors. The P2 receptor family is further classified into metabotropic P2Y receptors (i.e., P2Y_1,2,4,6,11–14_) and ionotropic P2X receptors (i.e., P2X1-7) (Abbracchio et al., 2006; Habermacher et al., 2016).

Pharmacological agonists and antagonists targeting purinergic receptors have gained widespread clinical interest and undergone clinical trials (Burnstock, 2017). P2X7 receptor (P2X7R) antagonists have been previously investigated in phase 2 clinical trials for treatment of inflammatory and autoimmune diseases, including chronic obstructive pulmonary disorder, rheumatoid arthritis and Crohn’s disease (Arulkumaran et al., 2011; Keystone et al., 2012). Recent advances in the development of neuro-permeable P2X7R antagonists have stimulated interest in the use of these compounds to treat neuroinflammatory and neuropsychiatric disorders (Chrovian et al., 2014; Burnstock and Knight, 2018; Bhattacharya and Ceusters, 2019). The P2X3 receptor (P2X3R) contributes to hypersensitivity of lung afferent sensory fibers that mediate cough initiation and phase 2 clinical trials have demonstrated that the P2X3R antagonist gefapixant (AF-219) reduces refractory chronic cough in afflicted patients by 75% (Weigand et al., 2012; Abdulqawi et al., 2015). Follow-up phase 3 clinical trials are currently underway to validate the use of gefapixant for treatment of refractory chronic cough (Muccino and Green, 2019).

Due to its ability to stimulate water transport across epithelial cell membranes following activation of calcium-dependent chloride channels, the P2Y_2_ receptor (P2Y_2_R) agonist diquafosol has undergone human clinical trials for the treatment of dry eye disease (DED) and is currently approved for human use in Japan and South Korea under the trade name Diquas (Tauber et al., 2004; Takamura et al., 2012; Koh, 2015). A similar P2Y_2_R agonist, denufosol, improved lung function relative to placebo in cystic fibrosis patients during phase 2 clinical trials, but failed to achieve its primary endpoints during phase 3 follow-up trials (Accurso et al., 2011). Notably, the FDA-approved anti-coagulant Plavix (clopidogrel), a P2Y_12_ receptor (P2Y_12_R) antagonist, was the 2^nd^ most prescribed drug in the world in 2010 and is currently on the World Health Organization’s List of Essential Medicines (Topol and Schork, 2011; Kishore et al., 2018). However, the therapeutic potential of targeting purinergic receptors has not been well-investigated in the context of human salivary dysfunction. In the salivary glands, several purinergic receptors are expressed and upregulated under pathological conditions, including SS (Schrader et al., 2005; Baldini et al., 2013), where their activation mediates inflammatory and immune responses (Baker et al., 2008; Khalafalla M.G. et al., 2017), as well as cell repair mechanisms (El-Sayed et al., 2014). In this review, we summarize the role of purinergic receptors in salivary gland function and highlight their potential as novel therapeutic targets to treat salivary gland dysfunction.

## The Role of P2 Receptors in Salivary Gland Function

**FIGURE 1:**
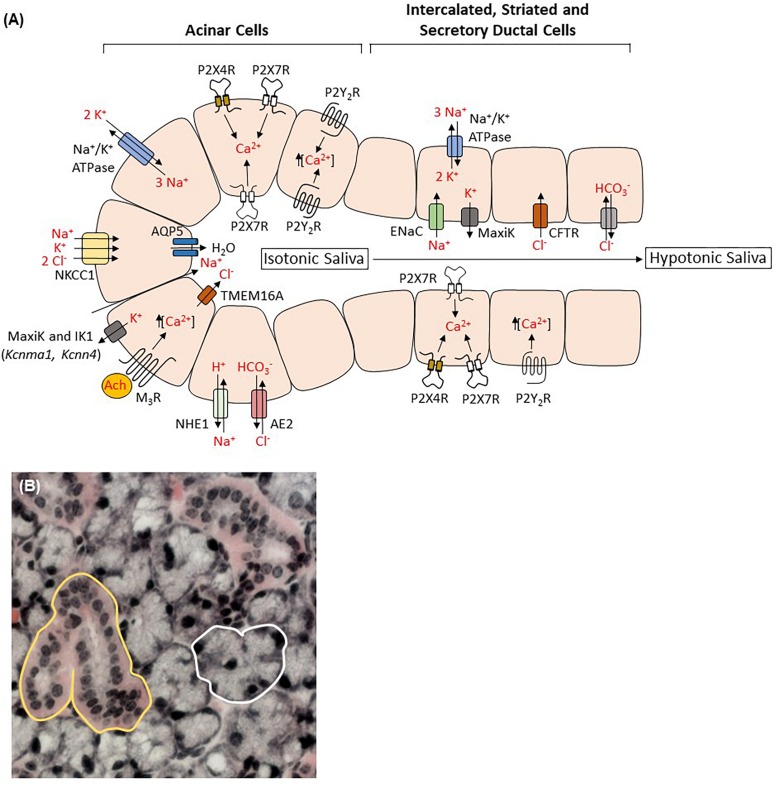
Salivary gland acinar and ductal cells contribute to saliva formation. **(A)** Activation of type 3 muscarinic receptors (M_3_R) by acetylcholine (Ach) increases release of calcium from intracellular stores and subsequent opening of the apical Ca^2+^-dependent chloride channel transmembrane member 16A (TMEM16A; also known as anoctamin-1) and the basolateral Ca^2+^-dependent potassium channels MaxiK (*Kcnma1*) and IK1 (*Kcnn4*), allowing Cl^–^ efflux into the luminal compartment and K^+^ efflux into the basolateral compartment to maintain membrane potential. The combined actions of the Na^+^/K^+^/2 Cl^–^ cotransporter NKCC1, the Na^+^/H^+^ exchanger NHE1 and the Cl^–^/HCO${}_{3}^{-}$ anion exchanger AE2 maintain the pool of intracellular Cl^–^ whereas the Na^+^/K^+^ ATPase generates the cellular Na^+^ and K^+^ gradients. Sodium influx down the negative electrochemical gradient into the luminal compartment is followed by water through aquaporin 5 (AQP5) water channels generating primary isotonic saliva. Modification of saliva by ductal cells involves exchanging sodium and chloride for potassium and bicarbonate through the combined actions of epithelial Na^+^ channels (ENaC), cystic fibrosis transmembrane conductance regulator (CFTR) channels, MaxiK channels and perhaps Cl^–^/HCO${}_{3}^{-}$ exchangers. The resulting hypotonic saliva is then secreted through ducts into the oral cavity. Functional P2X4, P2X7, P2Y_1_, and P2Y_2_ receptor expression has been demonstrated in both acinar and ductal cells where they may regulate secretory functions through nucleotide-induced Ca^2+^ signaling and modulation of membrane ion conductance. Available evidence suggests that P2X7 and P2Y_2_ receptors exist on both apical and basolateral membranes while P2X4 receptors are restricted to the basolateral compartment and P2Y_1_ receptor localization is undetermined. Importantly, P2 receptor expression in salivary gland tissue varies depending on species, isolation/culture methods and the presence of inflammatory stimuli, making definitive localization inexact. **(B)** Acinar (white) and ductal (yellow) cells outlined in a hematoxylin and eosin-stained section of a female C57BL/6 mouse submandibular gland.

The importance of saliva, as noted above, is clearly exemplified in individuals suffering from salivary gland hypofunction (Chambers et al., 2004; Atkinson et al., 2005; Meijer et al., 2009). In humans, whole unstimulated saliva is formed from the combined secretions of three pairs of major salivary glands, the submandibular (∼65%), parotid (∼20%) and sublingual (∼7%), along with numerous minor glands spread throughout the oral cavity that produce the remainder of saliva (<10%) (Humphrey and Williamson, 2001; de Almeida Pdel et al., 2008; Proctor, 2016). Upon stimulation, the parotid glands contribute the majority of total salivary secretions (Humphrey and Williamson, 2001; de Almeida Pdel et al., 2008; Proctor, 2016). Three basic cell types comprise the salivary glands: acinar epithelial cells that secrete the majority of the water and electrolytes in saliva, ductal cells that modify the electrolyte concentrations in the primary fluid and myoepithelial cells that provide contractile support for acinar cells (Martinez, 1987; Melvin et al., 2005; de Almeida Pdel et al., 2008; Proctor, 2016). Salivary acinar cells are either serous or mucous, whereas ductal cells are classified as intercalated, striated or excretory and the distribution of these cell types is dependent on species and type of gland (Melvin et al., 2005; de Almeida Pdel et al., 2008; Proctor, 2016). Along with the formation and modification of saliva, acinar and ductal cells also secrete important proteins, e.g., amylase and mucins from acinar cells (Boehlke et al., 2015; Frenkel and Ribbeck, 2015), kallikrein from ductal cells (Wong et al., 1983) and growth factors from both cell types (Masahiko et al., 2008), that are integral in maintaining the health of the oral cavity (Proctor, 2016). As shown in Figure 1, saliva formation is initiated in acinar cells by agonist-induced increases in intracellular Ca^2+^ levels, [Ca^2+^]_i_, that induce the opening of apical Ca^2+^-dependent Cl^–^ channels and basolateral Ca^2+^-dependent potassium channels, allowing Cl^–^ efflux into the luminal compartment and K^+^ efflux into the basolateral compartment to maintain membrane potential. The negative electrochemical gradient generated by increased luminal Cl^–^ levels is compensated by the influx of Na^+^ ions across tight junctions into the lumen leading to Na^+^Cl^–^ accumulation followed by water movement through water channels, predominately aquaporin-5 (Ma et al., 1999), thus forming saliva in its primary isotonic form. As saliva flows through the salivary gland ducts, electrolyte modification occurs, where Na^+^ and Cl^–^ ions are exchanged for K^+^ and HCO${}_{3}^{-}$ ions by ductal cells, creating saliva in its final hypotonic form (Martinez, 1987; Melvin et al., 2005; Lee et al., 2012; Ambudkar, 2014; Proctor, 2016). Several types of Ca^2+^ mobilizing receptors are expressed on acinar cells (i.e., muscarinic, α-adrenergic, substance P), however, stimulation of the G_q_ protein-coupled M3 muscarinic receptor (M_3_R) subtype by acetylcholine is accepted as the main receptor signaling pathway that promotes the increases in [Ca^2+^]_i_ necessary to enhance fluid secretion. Protein secretion from acinar and ductal cells is predominately mediated by activation of the β-adrenergic receptor (β-AR) and subsequent increases in cAMP (Melvin et al., 2005; Proctor, 2016). In addition to the canonical M_3_R and β-AR pathways, a mechanism of non-cholinergic, non-adrenergic-mediated salivary flow exists (Ekström et al., 1988; Ekström, 1999; Melvin et al., 2005). Because purinergic receptor activation can result in an increase in [Ca^2+^]_i_ in salivary gland cells, purinergic receptor-mediated saliva production may contribute to this non-canonical pathway (Turner et al., 1998b; Melvin et al., 2005; Aure et al., 2010; Bhattacharya et al., 2015).

In other exocrine tissues, purinergic receptor signaling has been shown to modulate secretory function of acinar and ductal cells through the induction of cellular ion fluxes and cross-talk with cholinergic signaling pathways (Burnstock and Novak, 2012; Hodges and Dartt, 2016). In the pancreas, acinar cells have little functional response to exogenously applied nucleotides (Novak et al., 2002), whereas ductal cells that secrete bicarbonate and isotonic fluid express numerous functional P2X and P2Y receptors (Hede et al., 1999). In response to stimulation by acetylcholine or secretin, pancreatic ductal cell secretion is mediated by the opening of luminal Cl^–^ channels, including Ca^2+^-activated Cl^–^ channels, as well as basolateral K^+^ channels to maintain driving force for ion transport (Novak, 2008). Therefore, the finding that extracellular ATP and UTP induce increases in [Ca^2+^]_i_ and modulate whole cell Cl^–^ and K^+^ conductance suggests a role for purinergic receptors in secretory regulation of pancreatic ductal cells (Christoffersen et al., 1998; Hede et al., 1999; Zsembery et al., 2000). Furthermore, studies have shown that cholinergic agonists induce ATP release from pancreatic acinar cells (Sorensen and Novak, 2001), as well as parotid and lacrimal gland cell preparations (Novak et al., 2010; Dartt and Hodges, 2011a), further supporting a role for purinergic signaling in the regulation of exocrine secretory function. In rat lacrimal gland acinar cells, extracellular nucleotide-induced protein secretion and [Ca^2+^]_i_ increases were inhibited by the cholinergic antagonist atropine (Dartt and Hodges, 2011a) whereas in rat parotid acinar cells extracellular nucleotides attenuated acetylcholine-induced [Ca^2+^]_i_ increases (Jorgensen et al., 1995; Fukushi, 1999). Although the nature of purinergic and cholinergic signaling interaction differs between exocrine tissues, these studies highlight the likely regulatory role of purinergic receptors in exocrine secretory function.

Ten years prior to the initial cloning and identification of P2 receptors, Gallacher (1982) presented the first evidence of P2 receptor activation in salivary glands. His studies demonstrated that ATP evoked a marked increase in membrane conductance, K^+^ efflux and amylase secretion in the mouse parotid gland, events similar to cholinergic- and adrenergic-mediated saliva secretion (Melvin et al., 2005; Proctor, 2016). McMillian et al. (1987) showed that high extracellular ATP concentrations increased [Ca^2+^]_i_ in rat parotid acinar cells, the signaling response that promotes saliva production (Melvin et al., 2005). Additional studies by the same group and others determined that the large ATP-induced rise in [Ca^2+^]_i_ was due to the influx of extracellular Ca^2+^ through a non-selective cation channel activated by the fully ionized form of ATP (i.e., ATP^4–^) (Soltoff et al., 1990; Dehaye, 1993; McMillian et al., 1993). The order of agonist potency for channel activation in these studies was determined to be BzATP > ATP > ATPγS = 2MeSATP; thus, the receptor was classified as P_2Z,_ now known as the P2X7 receptor (P2X7R) (Soltoff et al., 1990; Dehaye, 1993; McMillian et al., 1993). Thus, a physiological role for ATP in the Ca^2+^-dependent formation of saliva was proposed, particularly since ATP was known to be released as a co-transmitter from activated sympathetic and parasympathetic nerve fibers (von Kugelgen et al., 1994; Novak, 2003). During the ensuing years, especially following the cloning, expression and identification of cDNAs for a variety of P2 receptors in the early 1990s (Lustig et al., 1993; Webb et al., 1993; Nguyen et al., 1995; Surprenant et al., 1996), several groups confirmed the expression of P2X7R in salivary gland cells and also identified and functionally characterized the ionotropic P2X4 receptor (P2X4R) and metabotropic P2Y receptors, P2Y_1_R and P2Y_2_R, in these cells (Turner et al., 1999).

The P2X7R is a 595 amino acid protein that includes two transmembrane domains, intracellular carboxy and amino termini and a bulky hydrophilic extracellular loop with a cysteine rich region that forms disulfide bridges (McCarthy et al., 2019). It shares 40–50% amino acid homology with the other P2X receptors, but is structurally distinct in that its C-terminal tail extends for an additional 100–200 amino acids (North, 2002; Adinolfi et al., 2005; Sluyter, 2017). The P2X7R is activated by high extracellular ATP (eATP) concentrations (>100 μM) with brief stimulation (10–30 s) causing the depolarization of the plasma membrane due to the opening of a membrane cation channel that promotes the influx of Na^+^ and Ca^2+^ and the efflux of K^+^ (Weisman et al., 1984, 1989; Adinolfi et al., 2005). Sustained P2X7R activation induces the opening of a pore permeable to hydrophilic molecules up to 900 Da, and promotes production of reactive oxygen species (ROS), NLRP3 inflammasome-dependent IL-1β release, extensive plasma membrane blebbing and ultimately cell death (Weisman et al., 1984, 1989; Woods et al., 2012; Di Virgilio et al., 2017; Giuliani et al., 2017; Khalafalla M.G. et al., 2017). The P2X7R is widely expressed in diverse tissues, including hematopoietic cells (Feng et al., 2016), neurons (Miras-Portugal et al., 2017), glia (Stokes et al., 2015; Kaczmarek-Hajek et al., 2018), bone (Agrawal and Gartland, 2015), muscle (Fabbrizio et al., 2019), endothelium (Green et al., 2018), epithelium (Woods et al., 2012), and immune cells (Ferrari et al., 1997). In the exocrine pancreas, P2X7Rs have been shown to be primarily expressed in pancreatic ductal cells where they may contribute to secretory regulation through induction of cation fluxes and interaction with cholinergic signaling (Novak et al., 2010; Burnstock and Novak, 2012). Similarly, in lacrimal glands P2X7Rs mediate [Ca^2+^]_i_ increases, ERK1/2 activation, protein secretion and modulate both cholinergic and adrenergic receptor signaling pathways (Hodges et al., 2009; Dartt and Hodges, 2011a, b). After its initial characterization in rat parotid acinar cells (McMillian et al., 1987; Gibbons et al., 2001), P2X7R expression and function were reported to promote increases in [Ca^2+^]_i_ in rat submandibular acinar cells (Lee et al., 1997; Alzola et al., 2001), murine parotid (Li et al., 2003; Reyes et al., 2008; Bhattacharya et al., 2012) and submandibular acinar cells (Nakamoto et al., 2009) and human parotid acinar cells (Brown et al., 2004).

In addition to numerous studies defining its role in mediating inflammatory and immune responses in disease models (Savio et al., 2018; Cao et al., 2019; Zeng et al., 2019), including those pertaining to salivary glands (Woods et al., 2012; Khalafalla M.G. et al., 2017), there is evidence that P2X7Rs regulate salivary secretory function (Nakamoto et al., 2009; Novak et al., 2010; Pochet et al., 2013). Along with its ability to increase [Ca^2+^]_i_ due to calcium influx, P2X7R activation has been shown to inhibit mobilization of intracellular Ca^2+^ induced by muscarinic or substance P receptor agonists in rat submandibular acinar cells (Hurley et al., 1993; Metioui et al., 1996) and cholinergic mobilization of [Ca^2+^]_i_ was significantly increased in parotid acinar cells prepared from P2X7R-null (P2X7R^–/–^) mice (Novak et al., 2010). The mechanism of this inhibition is still unclear, but it does not appear to be due to interference with binding of the autonomic agonists to their receptors (Hurley et al., 1993). This observation was corroborated in an *ex vivo* murine submandibular gland (SMG) preparation, where co-stimulation with ATP and muscarinic receptor agonists had an inhibitory effect on the gland’s saliva production (Nakamoto et al., 2009). Further, in glands prepared from P2X7R^–/–^ mice the inhibitory effect of ATP on carbachol-induced saliva secretion was abolished, suggesting an inhibitory role for P2X7Rs in saliva production (Nakamoto et al., 2009). However, in this same study ATP or BzATP alone evoked fluid secretion in a time-dependent manner that was greatly reduced in glands from P2X7R^–/–^ mice, whereas carbachol alone induced similar saliva secretion in wild type and P2X7R^–/–^ glands. Similarly, another study found no significant difference in cholinergic-mediated whole saliva secretion in P2X7R^–/–^ mice compared to wild type (Pochet et al., 2007). In contrast, Novak et al. (2010) found that cholinergic-mediated whole saliva secretion was significantly decreased in P2X7R^–/–^ mice, as compared to wild type mice, and this was particularly evident in male mice. While the reasons for the disparities among these studies are unclear, they may be due to differences in the type of saliva collected (i.e., whole saliva vs. saliva from specific glands), methods of induction of saliva secretion, tissue specificity, sex, or mouse strain.

The P2X7R is also expressed in rat (Lee et al., 1997; Alzola et al., 1998) and mouse salivary ductal cells (Li et al., 2003; Pochet et al., 2007; Nakamoto et al., 2009), suggesting participation in the modification of the electrolyte content of saliva. Studies indicate no difference in [Na^+^] or [Cl^–^] in muscarinic agonist-induced whole saliva secreted in wild type compared to P2X7R^–/–^ mice, however the [K^+^] was elevated in P2X7R^–/–^ mouse whole saliva (Pochet et al., 2007). Since the majority of the K^+^ in saliva originates from ductal cells, it has been hypothesized that ATP released from acinar cells during exocytosis stimulates ductal P2X7Rs that regulate the activity of K^+^ channels located on the apical membrane (Liu et al., 1999; Bhattacharya et al., 2015). In addition to K^+^ modification, activation of P2X7Rs in ductal cells increases phospholipase A2-dependent secretion of arachidonic acid, a precursor of prostaglandin E2 (PGE_2_), and kallikrein (Alzola et al., 1998) into saliva (Pantano et al., 2019). Interestingly, cell lines of salivary origin exhibit low expression and function of P2X7R, which are enhanced following DNA demethylation (Shin et al., 2015).

Another P2X ionotropic receptor expressed in salivary acinar and ductal cells is the P2X4R (Turner et al., 1998b). Unlike the P2X7R’s requirement for activation by high eATP concentrations, P2X4Rs have nanomolar affinity for ATP (North, 2016; Suurvali et al., 2017) and were initially found to regulate the biphasic response to ATP in rat parotid gland cells (McMillian et al., 1993). The P2X4R is widely expressed in a variety of cell types, e.g., neurons and microglia (Ho et al., 2014), epithelium (Casas-Pruneda et al., 2009), and endothelium (Lv et al., 2015), and P2X4R expression in microglia is notable for the key role it plays in mediating neuropathic pain (Inoue, 2019). Although RT-PCR analysis has identified P2X4R expression in pancreatic acinar and ductal cells (Luo et al., 1999; Novak et al., 2002) and lacrimal gland acinar cells (Hodges et al., 2011; Kamada et al., 2012), its functional role in exocrine tissues remains largely unexplored. Physical interactions between P2X4Rs and P2X7Rs have been demonstrated, although the nature of this interaction remains controversial (Kopp et al., 2019). Some studies suggest that P2X4R and P2X7R subunits form heteromeric channels (Guo et al., 2007; Schneider et al., 2017), while others conclude that P2X4 and P2X7 receptors interact in their respective homotrimeric form (Nicke, 2008; Boumechache et al., 2009; Antonio et al., 2011). Furthermore, P2X4R expression has been localized to lysosomal membranes, whereas P2X7Rs primarily reside at the plasma membrane (Guo et al., 2007; Huang et al., 2014). Nevertheless, studies have also demonstrated functional evidence for P2X4R/P2X7R interactions (Guo et al., 2007; Kawano et al., 2012; Perez-Flores et al., 2015). In salivary epithelium, P2X4Rs modulate P2X7R-mediated ion flow and ethidium bromide dye uptake (Casas-Pruneda et al., 2009), suggesting a functional interaction that regulates physiological processes, including plasma membrane ion channel function and pore formation. Importantly, the interaction between these two purinergic receptors results in a decreased sensitivity to ATP, as compared to the P2X4R or P2X7R alone, suggesting the formation of heteromeric channels with novel functional and pharmacological properties (Casas-Pruneda et al., 2009).

While the contribution of P2X4R activation to physiological saliva production has not been explored, *ex vivo* murine SMG preparations from P2X7R^–/–^ mice exhibit weak ATP-induced saliva secretion that could be attributed to P2X4R activation (Nakamoto et al., 2009). As seen previously with muscarinic or adrenergic receptor activation (Baldys-Waligorska et al., 1987; Yoshimura and Hiramatsu, 1998; Tanimura et al., 1999; Bruce et al., 2002), co-stimulation of β-adrenergic receptors and P2X7Rs or P2X4Rs enhanced the influx of Ca^2+^ in mouse parotid acinar cells, as compared to activation of either receptor alone (Bhattacharya et al., 2015). In contrast, studies using human parotid acinar cells found this co-stimulatory effect only between the P2X4R and β-adrenergic receptor (Brown et al., 2004). Taken together, the expression of both P2X7Rs and P2X4Rs in salivary glands supports the idea that they are involved in the interplay between canonical and non-canonical signaling pathways that regulate saliva flow and composition and their involvement is likely dependent on their tissue localization (i.e., basal vs. apical) in polarized acinar and/or ductal epithelial cells (Bhattacharya et al., 2012, 2015).

The metabotropic P2Y_1_ receptor (P2Y_1_R), formerly known as the P_2T_ receptor, has been identified and cloned (Webb et al., 1993; Baranska et al., 2017) and has features typical of G protein-coupled receptors, i.e., an extracellular N-terminus and an intracellular C-terminus, seven hydrophobic transmembrane regions, three extracellular loops and three intracellular loops (von Kugelgen and Hoffmann, 2016). The P2Y_1_R has a distinctive rank order of agonist potencies (i.e., 2-methylthio-ADP > ADP > ATP) and its activation induces canonical Gα_q_ signaling leading to phospholipase C activation and generation of the second messengers inositol 1, 4, 5-trisphosphate (IP_3_) and diacylglycerol that increase [Ca^2+^]_i_ and protein kinase C (PKC) activity, respectively (von Kugelgen and Wetter, 2000; Abbracchio et al., 2006; Baranska et al., 2017; von Kugelgen, 2019). Additionally, P2Y_1_R activation stimulates metalloprotease-dependent transactivation of the epidermal growth factor receptor (EGFR) (Buvinic et al., 2007) and mitogen-activated protein kinase (MAPK) activity through activation of phosphatidylinositol 3-kinase, Src kinase and PKC (Sellers et al., 2001; Baranska et al., 2017). The P2Y_1_R is widely distributed in mammalian tissues and is involved in many physiological and biochemical responses, such as platelet aggregation (Fabre et al., 1999), pain sensation (Barragan-Iglesias et al., 2015), vasodilation (Zerr et al., 2011), bone remodeling (Orriss et al., 2011), and osmotic volume regulation (Grosche et al., 2013). In exocrine tissues, immunofluorescence and RT-PCR analyses provide evidence of P2Y_1_R expression in pancreatic ductal cells where P2Y_1_R agonists also induce [Ca^2+^]_i_ increases (Luo et al., 1999; Coutinho-Silva et al., 2001). However, the role of P2Y_1_Rs in exocrine pancreas function has been unexplored. Likewise, P2Y_1_R expression has been demonstrated in lacrimal acinar cells and myoepithelial cells by RT-PCR, immunofluorescence and measurement of P2Y_1_R agonist-induced [Ca^2+^]_i_ increases, but further functional analyses are lacking (Ohtomo et al., 2011). Interestingly, the P2Y_1_R has been used as a surrogate cell-surface marker for the nuclear protein pancreatic duodenal homeobox 1 (PDX1) to isolate progenitor-like ductal cells from human pancreatic tissues, although no functional role for P2Y_1_Rs was investigated (Qadir et al., 2018). In contrast, studies on endocrine pancreas function suggest a role for P2Y_1_Rs in mediating insulin secretion from β cells (Leon et al., 2005; Petit et al., 2009). The P2Y_1_R is also involved in tissue development, as was first described in chick embryos (Meyer et al., 1999; Meyer et al., 2001) and more recently in the developing brain (Huang et al., 2019). In the developing rat salivary gland, it was observed that acinar cells prepared from immature glands of 1 day-old pups had a robust [Ca^2+^]_i_ response to P2Y_1_R agonists, whereas acini prepared from adult rat salivary glands had no response (Park et al., 1997). Interestingly, P2Y_1_R mRNA expression remained the same at all ages in rats, suggesting that the loss of the P2Y_1_R-mediated [Ca^2+^]_i_ response may be due to age-dependent alterations in intracellular G protein coupling (Park et al., 1997). A subsequent study using rat SMG acinar and ductal cell preparations confirmed the age-dependent reduction in P2Y_1_R-mediated increases in [Ca^2+^]_i_ and, similarly, found unchanged P2Y_1_R expression levels at all ages (Baker et al., 2006). This study further demonstrated that P2Y_1_R-mediated activation of the MAPKs, extracellular signal-regulated kinases 1 and 2 (ERK1/2), was consistent in rats of all ages, indicating that ERK1/2 activation is independent of P2Y_1_R-mediated changes in [Ca^2+^]_i_. Western analysis and assays of GTPγ^35^S binding to G proteins determined that the age-dependent decrease in P2Y_1_R activity in rat SMG cells was due to both decreased expression of the 52 kDa Gα_14_ protein and differential coupling of P2Y_1_Rs to Gα_q/11_ with age (Baker et al., 2006). These studies suggest that P2Y_1_Rs use diverse mechanisms for coupling to multiple G proteins that regulate a variety of physiological responses during development. To date, these findings have not been confirmed in salivary glands of mice, but with the availability of P2Y_1_R-null mice, it would be of interest to assess the role of this receptor in salivary gland morphology and function during development.

The P2Y_2_R (formerly known as the P_2U_ receptor), equipotently activated by ATP or UTP (EC_50_ ∼ 2 μM), is the only other known Gα_q_-coupled purinergic receptor identified in salivary glands (Turner et al., 1998b, 1999) and has been cloned and functionally characterized in mice and humans (Erb et al., 1993; Lustig et al., 1993; Parr et al., 1994). Similar to the P2Y_1_R, P2Y_2_R activation induces canonical Gα_q_ signaling leading to increases in [Ca^2+^]_i_ and PKC activation, and the P2Y_2_R is expressed in numerous cell and tissue types, e.g., neurons (Peterson et al., 2013), epithelium (Shishikura et al., 2016; Wu et al., 2017), endothelium (Seye et al., 2003) and immune cells (Idzko et al., 2014; Woods et al., 2018), where it modulates a variety of cellular responses, including neurotransmission (Zhang and Li, 2019), proliferation (Shen et al., 2004), cell migration (Bagchi et al., 2005), cytoskeletal rearrangements (Liao et al., 2007), and ion fluxes (Murakami et al., 2004). The diversity of cellular responses mediated by P2Y_2_Rs is due, in part, to unique structural features enabling activation of multiple signal transduction pathways. In addition to canonical Gα_q_ signaling (Parr et al., 1994), the P2Y_2_R contains a motif typically found in extracellular matrix proteins, i.e., an Arg-Gly-Asp (RGD)-sequence, in its first extracellular loop that binds to α_v_β_3_/β_5_ integrins to activate G_o_ and G_12_ proteins, enhance MAPK (ERK1/2) phosphorylation and regulate ATP- and UTP-induced cell chemokinesis and chemotaxis (Erb et al., 2001; Bagchi et al., 2005; Wang et al., 2005; Liao et al., 2007). Within the intracellular C-terminus of the P2Y_2_R, Src-homology-3 (SH3) binding domains (PXXP) enable the P2Y_2_R to bind and activate the tyrosine kinase Src, enabling nucleotide-induced, Src-dependent transactivation of growth factor receptors and downstream MAPKs that regulate cell proliferation and migration (Liu et al., 2004; Seye et al., 2004). Additionally, interaction of the P2Y_2_R C-terminus with the actin-binding protein filamin-A contributes to cell migration and Rho GTPase-mediated cytokine release (Yu et al., 2008; Seye et al., 2012). The P2Y_2_R also mediates the proprotein convertase furin-dependent activation of metalloproteases, i.e., a disintegrin and metalloproteinase 10 and 17 (ADAM10/17), to cleave transmembrane proteins (Camden et al., 2005), thereby releasing EGFR/ERB ligands that promote Src-independent EGFR activation (Ratchford et al., 2010). These diverse P2Y_2_R signaling pathways have been implicated in a number of pathologies, including Alzheimer’s disease (Ajit et al., 2014), cardiovascular disease (Chen et al., 2017), cancer (Hu et al., 2019), SS (Woods et al., 2018), and hantavirus cardiopulmonary syndrome (Bondu et al., 2018), as well as processes such as wound healing (Jin et al., 2014) and tissue regeneration (El-Sayed et al., 2014).

In exocrine tissues such as the lacrimal gland, RT-PCR and immunohistochemical analyses have identified P2Y_2_R expression in acinar and ductal cells (Kamada et al., 2012; Tanioka et al., 2014). While no functional response to the P2Y_2_R agonist UTP was observed in lacrimal acinar cells (Kamada et al., 2012), cultured lacrimal gland myoepithelial cells do exhibit increased [Ca^2+^]_i_ in response to extracellular UTP suggesting the presence of P2Y_2_ or P2Y_4_ receptors (Ohtomo et al., 2011). In the exocrine pancreas, RT-PCR and immunohistochemical analyses indicate that P2Y_2_Rs are expressed in both pancreatic acini (Novak et al., 2002) and ductal cells (Hede et al., 1999; Luo et al., 1999; Coutinho-Silva et al., 2001), although very few pancreatic acinar cells show functional responses to extracellular ATP or UTP (Novak et al., 2002). In pancreatic ductal cells, P2Y_2_R-mediated increases in [Ca^2+^]_i_ altered whole-cell K^+^ conductance (Hede et al., 1999), likely through modulation of Ca^2+^-activated K^+^ channels (Hede et al., 2005), suggesting a role in the regulation of ductal fluid flow and Cl^–^/HCO${}_{3}^{-}$ levels. Studies with pancreatic ductal cell lines have also shown that the P2Y_2_R agonists ATP and UTP increase membrane Cl^–^ conductance through the opening of Ca^2+^-dependent Cl^–^ channels (Galietta et al., 1994; Chan et al., 1996; Zsembery et al., 2000). The ability of P2Y_2_Rs to induce chloride secretion and subsequent fluid flow across epithelial cell membranes led to investigation of the P2Y_2_R as a therapeutic target for cystic fibrosis (Weisman et al., 1998; Kellerman et al., 2002; Lazarowski and Boucher, 2009). By stimulating Ca^2+^-dependent Cl^–^ secretion, topical application of the selective P2Y_2_R agonist diquafosol has been shown to promote tear secretion and is currently being used to treat DED (Jacobson and Civan, 2016).

In 1991, the P2Y_2_R was first identified in a cell line of salivary gland origin, human salivary gland (HSG) cells, where it was shown to mediate UTP-induced IP_3_ production and increases in [Ca^2+^]_i_ and plasma membrane K^+^ transport (Yu and Turner, 1991). A subsequent study determined that exposure of HSG cells to UTP potentiated a regulatory volume decrease (RVD) after hypotonic stress, suggesting that activation of P2Y_2_Rs provides the driving force for net Cl^–^ efflux that enables the cells to rapidly restore their volume (Kim et al., 1996), a response that occurs during salivary secretion (Melvin et al., 2005). In 1998, it was shown that simian virus 40-transformed salivary cell lines from rat SMG and parotid glands (Quissell et al., 1998), unlike HSG cells, were suitable for Ussing chamber studies due to their ability to form polarized cell monolayers (Turner et al., 1998a). Using the polarized rat parotid cell line Par-C10 in a Ussing chamber, transepithelial resistance measurements determined that functional P2Y_2_R expression was localized to the apical membrane, consistent with its localization in other epithelium (Hwang et al., 1996; Chan et al., 1997; Yang et al., 2009), and its activation by UTP increased an anion (${\text{Cl}}^{-}/{\text{HCO}}_{3}^{-}$)-dependent change in short-circuit current (I_sc_) (Chan et al., 1996, 1997; Clarke et al., 1999). Taken together, these results suggest that expression of P2Y_2_Rs on salivary gland epithelium may contribute to saliva secretion; however, subsequent studies with freshly isolated salivary acinar cells showed little evidence of P2Y_2_R expression or activity under steady-state conditions (Turner et al., 1997; Ahn et al., 2000; Schrader et al., 2005). Moreover, carbachol-stimulated whole saliva secretion in P2Y_2_R-null mice (P2Y_2_R^–/–^) is unchanged compared to wild type mice (Woods et al., 2018), suggesting that P2Y_2_Rs do not contribute to overall fluid secretion. Earlier studies demonstrated UTP-induced Cl^–^ fluxes in rat salivary duct cells (Lee et al., 1997; Zeng et al., 1997) with one study suggesting that P2Y_2_R expression on striated ducts regulates CFTR activity (Ishibashi et al., 2008), thereby possibly modifying the ionic content of saliva.

## The Role of P2 Receptors in Salivary Gland Inflammation

The contribution of P2 receptors to physiological salivary gland function is predicated on the presence of endogenous agonists (i.e., extracellular nucleotides) in sufficient concentrations to activate their cognate receptors, as is the case when ATP is co-released with neurotransmitters from sympathetic and parasympathetic nerves (von Kugelgen et al., 1994; Novak, 2003). In exocrine tissues such as the pancreas and lacrimal glands, ATP is released in response to stimulation by physiological agonists such as acetylcholine and cholecystokinin-8 (Sorensen and Novak, 2001; Yegutkin et al., 2006; Novak et al., 2010; Dartt and Hodges, 2011a). Additionally, measurable amounts of ATP are present in rat saliva induced by intraperitoneal pilocarpine administration (Ishibashi et al., 2008). However, the concentration of extracellular nucleotides is tightly regulated under physiological conditions and maintained in the low μM range by ectonucleotidases (Pellegatti et al., 2008; Di Virgilio et al., 2018), such as the nucleoside triphosphate diphosphohydrolase ENTPD1 (CD39) and related family members (Deaglio and Robson, 2011; Zimmermann et al., 2012). Using conventional luciferin/luciferase luminescence measurements or cell-based biosensors, the concentration of extracellular ATP released from pancreatic acinar or β cells has been measured at ∼10–25 μM (Hazama et al., 1998; Sorensen and Novak, 2001), although *in vivo* measurement of absolute extracellular nucleotide concentrations is an active area of research (De Marchi et al., 2020). However, during periods of inflammation or other cellular stresses, such as hypoxia in the tumor microenvironment, extracellular ATP levels have been shown to exceed 100 μM and are likely much higher in the context of the confined pericellular space (Pellegatti et al., 2008; Joo et al., 2014; Di Virgilio et al., 2018; De Marchi et al., 2019). Immune and apoptotic cells release ATP through connexin and pannexin hemichannels during inflammatory responses and uncontrolled release of intracellular ATP pools can also occur during cell necrosis (Eltzschig et al., 2006; Chekeni et al., 2010). Mounting evidence also suggests that connexin 43-mediated ATP release from γ-irradiated cells causes the radiation-induced bystander effect where adjacent, non-irradiated cells exhibit physiological responses mediated by P2 receptors (Tsukimoto et al., 2010; Ohshima et al., 2012; Tsukimoto, 2015; Kojima et al., 2017). Interestingly, the ionotropic P2X7 receptor also has been shown to mediate ATP release (Suadicani et al., 2006; Ohshima et al., 2010), likely through its sustained activation that leads to membrane depolarization and pore formation (Dahlquist et al., 1974; Weisman et al., 1984; Buisman et al., 1988), and P2X7R blockade has been shown to attenuate ionizing radiation (IR)-induced ATP release from salivary acinar cells (Gilman et al., 2019). Recognizing that salivary gland inflammation and radiation exposure, two common sources of salivary gland dysfunction, promote the release of extracellular nucleotides and subsequent P2 receptor activation, defining the role of P2 receptors in salivary gland pathophysiology has been an area of intense interest.

In addition to its role as an ion channel, activation of the P2X7R initiates signaling cascades that produce pro-inflammatory cytokines (e.g., IL-1β, IL-18, IL-6, IL-8, and TNF-α) to enable antigen-presenting cells to initiate innate immune responses (Ferrari et al., 1997; Solini et al., 1999; Mehta et al., 2001; Lister et al., 2007; Shieh et al., 2014). In salivary epithelium, our group has shown that P2X7R activation with ATP or BzATP triggers apoptotic and pro-inflammatory cell responses, including increases in caspase-1 and caspase-3 activity and immune cell infiltration into wild type, but not P2X7R^–/–^, mouse SMGs (Woods et al., 2012). Also, P2X7R activation in salivary epithelium was found to induce the assembly of the NLRP3 inflammasome multiprotein complex and the subsequent release of IL-1β, a response that was dependent on K^+^ efflux, production of ROS and functional heat shock protein 90 (Khalafalla M.G. et al., 2017). P2X7R activation also has been shown to mediate the protease-dependent release of α-fodrin (Woods et al., 2012), a putative autoantigen associated with SS (Miyazaki et al., 2005), through a mechanism that requires caspase-3 and calpain enzymatic activities (Hwang et al., 2009b). P2X7R activation induces membrane blebbing, an early indicator of cell apoptosis, in salivary epithelial cells isolated from wild type, but not P2X7R^–/–^, mice (Woods et al., 2012). The mechanism of P2X7R-mediated membrane blebbing was shown to require sustained elevation of [Ca^2+^]_i_, activation of the ROCK I signaling pathway and phosphorylation of myosin light chain, but does not involve caspase-3 activation (Hwang et al., 2009a).

There are increasing lines of evidence that P2X7R-induced pro-inflammatory responses are modulated by the P2X4R as well. In immune cells, P2X4Rs have been shown to modulate P2X7R-induced IL-1β release and dye uptake through interaction with the P2X7R C-terminus and P2X4R antagonism abolished P2X7R-induced Ca^2+^ influx and IL-1β and IL-18 release (Sakaki et al., 2013). In gingival epithelial cells, P2X7Rs, P2X4Rs and pannexin-1 hemichannels were all required for ATP-induced ROS production, NLRP3 inflammasome activation and IL-1β release (Hung et al., 2013). These cellular mechanisms may also be important in IL-1β release from salivary epithelium, where P2X4Rs have been shown to modulate P2X7R-mediated ion flow and pore formation (Casas-Pruneda et al., 2009).

In rodent salivary glands, P2Y_2_R expression is negligible under physiological conditions. Interestingly, freshly dispersed salivary epithelial cells significantly upregulated P2Y_2_R expression and activity as a function of time when placed in culture (Turner et al., 1997; El-Sayed et al., 2014), consistent with a possible role for P2Y_2_R in the cellular response to stress. P2Y_2_R upregulation also occurs in the *in vivo* ductal ligation model of salivary gland inflammation and fibrosis (Ahn et al., 2000) and has been similarly seen in other *in vivo* models of stress and inflammation, i.e., intestinal inflammation (Grbic et al., 2008), rat vascular neointima formation after balloon angioplasty (Seye et al., 1997), collared rabbit carotid arteries (Seye et al., 2002), glomerulonephritis (Rennert et al., 2018), myocardium of rats with congestive heart failure (Granado et al., 2015) and mouse models of the autoimmune disease SS (Schrader et al., 2005; Woods et al., 2018). IL-1β has been previously shown to induce P2Y_2_R upregulation (Kong et al., 2009; Peterson et al., 2013), likely through binding of NF-κB p65 to the *P2Y_2_R* promoter region that has been demonstrated to mediate inflammation-induced P2Y_2_R upregulation in human intestinal epithelial cells (Degagne et al., 2009). Taken together, these studies suggest that ATP released from stressed cells during inflammation activates P2X7Rs to induce the release of IL-1β and other cytokines. Subsequent activation of IL-1 receptors by IL-1β in surrounding cells induces P2Y_2_R upregulation and further downstream responses to ATP and UTP. In this way, the release of a single alarmin (e.g., ATP or UTP) in response to cellular stress can locally modulate a wide range of signaling pathways to fine-tune the tissue response to inflammatory stimuli.

In HSG cells, UTP-induced activation of P2Y_2_Rs has been shown to regulate localized immune responses and the binding of immune cells through the upregulation of the cell adhesion molecule VCAM-1 via an EGFR-dependent mechanism (Baker et al., 2008). Furthermore, P2Y_2_R activation has been shown to stimulate the production and secretion of pro-inflammatory lymphotoxin-α (LT-α), a member of the tumor necrosis factor family of cytokines that is required for the development of lymphoid tissues and mediates interactions between immune cells (Shen et al., 2010, 2013), suggesting multiple mechanisms whereby P2Y_2_Rs regulate localized immune responses relevant to salivary gland inflammation (Seye et al., 2012; Qian et al., 2016; Woods et al., 2018).

## P2 Receptors in Sjögren’s Syndrome

A number of autoimmune inflammatory diseases are reported to impact the function of salivary glands, including rheumatoid arthritis (Nagler et al., 2003; Helenius et al., 2005; Zalewska et al., 2011), systemic lupus erythematosus (SLE) (Leite et al., 2015) and diabetes mellitus (Moore et al., 2001). One of the major causes of salivary gland dysfunction is chronic inflammation associated with the autoimmune disease SS, the 2^nd^ most common autoimmune rheumatic disease in the U.S., in which unresolved inflammation of the salivary and lacrimal glands contributes to tissue degeneration and subsequent loss of function (Helmick et al., 2008; Vivino, 2017). Clinical classification criteria for primary SS (pSS) in the absence of other autoimmune diseases include the presence in blood serum of anti-Ro/SSA and anti-La/SSB autoantibodies to their intracellular antigens, increased corneal staining using fluorescein dye (ocular staining score ≥ 5), decreased tear (Schirmer’s test ≤ 1 mm/min) and saliva (≤ 0.1 ml/min) flow rates and the presence of focal lymphocytic sialadenitis (focus score ≥ 1 foci/4 mm^2^) in minor salivary gland biopsies (Shiboski et al., 2017). During SS pathogenesis, T and B cells (van Woerkom et al., 2005; Daridon et al., 2006), dendritic cells (Ozaki et al., 2010; Zhao et al., 2016), and macrophages (Manoussakis et al., 2007) accumulate in the salivary glands where, along with salivary gland epithelial cells, they produce numerous pro-inflammatory cytokines, including IFN-γ, B cell-activating factor, TNF-α, IL-1β, IL-6 and IL-18, which initiate pro-inflammatory immune responses that ultimately degenerate the salivary glands (Hulkkonen et al., 2001; Willeke et al., 2003; Daridon et al., 2007; Sakai et al., 2008; Nezos et al., 2015). Additionally, SS patients produce high levels of immunoglobulins and autoantibodies besides anti-Ro/SSA and anti-La/SSB (Nardi et al., 2006; Suresh et al., 2015), including anti-α-fodrin (Watanabe et al., 1999; Miyazaki et al., 2005), RF (rheumatoid factor) (Müller et al., 1989; Huo et al., 2010) and other autoantibodies (Ramos-Casals et al., 2006; Shen et al., 2014; Suresh et al., 2015) that have been previously reported to activate intrinsic and extrinsic apoptotic pathways in salivary gland cells (Sisto et al., 2006; Lisi et al., 2007). Furthermore, anti-muscarinic receptor-3 autoantibodies that inhibit saliva production and aquaporin translocation to the plasma membrane (Bacman et al., 1996; Dawson et al., 2006) have been identified in the blood serum of SS patients. Taken together, these data suggest that chronic auto-inflammatory responses along with autoantibody-induced reductions in saliva and tear production and increased salivary acinar cell apoptosis contribute to pSS pathogenesis that ultimately leads to salivary gland dysfunction and fibrosis as well as systemic pathologies (i.e., chronic fatigue, lymphoma development, and secondary autoimmune manifestations).

Previous studies have demonstrated that the expression of *P2X7R*, *caspase-1*, *IL-1β*, *IL-18* and components of the NLRP3 inflammasome multiprotein complex are significantly increased in labial salivary gland biopsies from SS patients, which positively correlates with salivary gland focus score (# of mononuclear cell foci/4 mm^2^ tissue area) (Baldini et al., 2013, 2017). Furthermore, these studies found that when SS patients were stratified based on the presence of anti-Ro/SSA autoantibodies, the increased expression of *P2X7R* and NLRP3 inflammasome components was even more pronounced in seropositive cohorts compared to seronegative cohorts (Baldini et al., 2013, 2017). Subsequent immunofluorescence analysis indicated that P2X7R expression in SS salivary gland biopsies co-localized with the acinar epithelial cell marker aquaporin 5, rather than immune cell markers, suggesting that P2X7Rs on salivary gland epithelium contribute to SS pathogenesis through a process termed autoimmune epithelitis (Mitsias et al., 2006; Baldini et al., 2017). Additionally, this prospective study of 147 SS patients over ∼5 years found that those who eventually developed mucosa-associated lymphoid tissue non-Hodgkin lymphoma (MALT NHL), a serious complication of SS, had significantly higher labial salivary gland *P2X7R* expression at the time of SS diagnosis compared to non-lymphoma SS patients, suggesting that P2X7R expression may be a useful biomarker for MALT NHL development (Baldini et al., 2017). In an analysis of *P2X7R* functional polymorphisms in 114 SS patients and 136 non-SS controls, the frequency of a single nucleotide polymorphism in exon 13 (A1405G, rs2230912) was significantly increased in seropositive SS patients, as compared to control subjects (Lester et al., 2013). As determined by ATP-induced ethidium bromide uptake to detect P2X7R activation in isolated peripheral blood lymphocytes, the P2X7R A1405G polymorphism was found to be a gain-of-function mutation that was suggested to be a risk factor for seropositive SS in the absence of other SS-associated human leukocyte antigen risk alleles. However, this A1405G association failed to be replicated in a larger patient cohort (Lester et al., 2013).

Antagonism of the P2X7R, whose encoding gene is located within a mapped SLE susceptibility region on chromosome 12 (Elliott et al., 2005), has been investigated as a potential treatment for several inflammatory diseases, including SLE (Turner et al., 2007; Taylor et al., 2009), rheumatoid arthritis (Arulkumaran et al., 2011) and chronic obstructive pulmonary disease (Lucattelli et al., 2011). Due to its increased expression in salivary gland biopsies from SS patients (Baldini et al., 2013) and its reported role in the activation of pro-inflammatory responses in salivary epithelium (Woods et al., 2012), the P2X7R has emerged as an appealing therapeutic target to treat SS. Our group reported that *in vivo* inhibition of P2X7Rs using the competitive antagonist A-438079 significantly reduced sialadenitis and improved carbachol-induced saliva flow in the NOD.H-2^h4^, CD28^–/–^, IFNγ^–/–^ murine model of SS-like salivary gland autoimmune exocrinopathy (Khalafalla M.G. et al., 2017). P2X7R antagonism also significantly reduced salivary gland expression of immunoactive molecules known to be upregulated in salivary gland biopsies isolated from SS patients, including IL-1β, ICAM, VCAM, E-selectin, CD80, and CD86 (Tsunawaki et al., 2002; Khalafalla M.G. et al., 2017). Taken together, these studies suggest that the P2X7R represents a promising target for therapeutic intervention in salivary gland inflammation.

Previous studies have demonstrated that the P2Y_2_R is upregulated in major salivary glands of several mouse models of SS, including NOD.B10 (Schrader et al., 2005), IL-14α transgenic (IL-14αTG) (Woods et al., 2018) and C57BL/6-NOD.*Aec1Aec2* mice (unpublished observations). It was recently reported by our group that P2Y_2_R expression was increased in both SMG epithelium and SMG-infiltrating B cells in aged IL-14αTG mice with SS-like disease and genetic deletion of the P2Y_2_R attenuated both B and T cell infiltration of the salivary glands (Woods et al., 2018). Additionally, attenuated sialadenitis following P2Y_2_R deletion correlated with significantly reduced levels of LT-α in salivary gland epithelial cells and infiltrating immune cells, suggesting that P2Y_2_R-mediated LT-α expression contributes to salivary gland inflammation in IL-14αTG mice (Woods et al., 2018). Interestingly, LT-α levels are increased in the saliva, serum and salivary glands of SS patients, as compared to healthy individuals (Shen et al., 2010; Teos et al., 2015), and blockade of the LT-α receptor has been shown to reduce sialadenitis and improve the secretory function of the salivary gland in the IL-14αTG and NOD mouse models of SS (Gatumu et al., 2009; Shen et al., 2013). Lastly, unpublished observations from our lab indicate that expression of the *P2Y_2_R* is increased in salivary gland-infiltrating B cells in NOD.H-2^h4^, CD28^–/–^, IFNγ^–/–^ mice, as compared to B cells isolated from salivary glands of C57BL/6 control mice, and intraperitoneal administration of the selective P2Y_2_R antagonist AR-C118925 significantly attenuates sialadenitis and restores salivary gland function. In summary, these studies highlight the significant contributions of purinergic receptors to salivary gland inflammation and demonstrate their therapeutic potential for the treatment of human pro-inflammatory autoimmune diseases.

## P2 Receptors in Radiation-Induced Hyposalivation

Radiation-induced salivary gland dysfunction is a common unintended side effect of radiotherapy in head and neck cancer patients, which causes xerostomia and hyposalivation that affects > 95% of these patients, > 73% of whom continue to suffer from months to years after completion of the radiotherapy (PDQ Supportive and Palliative Care Editorial Board, 2002; Dirix et al., 2006; Jensen et al., 2010; Pinna et al., 2015). Head and neck cancer patients routinely receive fractionated radiation treatment where the tumor region receives high radiation doses while salivary gland sparing techniques attempt to limit the radiation dose to 2 Gy/day (Eisbruch et al., 1999; Grundmann et al., 2009; Pfister et al., 2015). It is estimated that the tolerance dose for a 50% complications rate (TD50) for the parotid and submandibular glands is 28.4 and 39 Gy, respectively (Eisbruch et al., 1999; Li et al., 2007; Murdoch-Kinch et al., 2008). A number of factors including tumor grade, lymph node involvement and location of the tumor create scenarios where salivary gland sparing is not feasible and the tissue is exposed to higher radiation doses. Consequently, chronic hyposalivation and changes in the saliva electrolyte composition occur along with a reduction in pH that leads to alterations in oral microbial flora, increased incidence of dental carries and oral infections and difficulties with swallowing, digestion, and speech (Hu et al., 2013; Pinna et al., 2015).

Several groups have utilized rodent models to demonstrate that acute hyposalivation occurs immediately after IR, before the onset of overt gland damage, which is associated with sustained increases in the [Ca^2+^]_i_ (Coppes et al., 2005; Liu et al., 2013, 2017; Ambudkar, 2018). In contrast, chronic IR-induced salivary dysfunction results from ROS production, increased caspase-3 activity, disruption of store-operated Ca^2+^ entry (SOCE), cytoskeletal rearrangements, acinar cell apoptosis, sialadenitis and replacement of normal parenchyma with fibrotic tissue (Coppes et al., 2001; Radfar and Sirois, 2003; Teymoortash et al., 2005; Muhvic-Urek et al., 2006; Avila et al., 2009; Liu et al., 2013, 2017; Wong et al., 2018). One of the early responses to IR is impairment of muscarinic receptor signaling (Coppes et al., 2000, 2005; Konings et al., 2005) required for saliva formation and aquaporin channel activity required for fluid secretion (Takagi et al., 2003). Furthermore, Avila et al. (2009), have demonstrated that radiation also causes a significant reduction in saliva-secreting acinar cells due to p53-dependent apoptosis. Thus, the overall mechanism of radiation-induced salivary gland hypofunction likely involves perturbations in muscarinic receptor signaling, apoptosis of saliva-producing acinar cells and irreversible tissue damage.

The P2X7R is highly expressed in salivary epithelium where its activation induces responses associated with IR-induced hyposalivation, including ROS production, caspase-3 activity, prostaglandin E_2_ and ATP release, NLRP3 inflammasome activation with IL-1β release and salivary gland cell apoptosis (Woods et al., 2012; Khalafalla M.G. et al., 2017; Gilman et al., 2019). Thus, we recently explored the role of P2X7R activation in γ-radiation-induced hyposalivation. IR exposure induced ATP release from wild type mouse parotid gland epithelial cells (PGECs) that was attenuated by the P2X7R antagonist A-438079 and in PGECs isolated from P2X7R^–/–^ compared to wild type mice (Gilman et al., 2019). Furthermore, systemic administration of A-438079 in γ-irradiated wild type mice conferred significant radioprotection to salivary glands and maintained saliva flow rates similar to non-irradiated mice at 3 and 30 days post-IR. This study also demonstrated that PGE_2_ is secreted from wild type PGECs following γ-radiation that was reduced in P2X7R^–/–^ PGECs or following A-438079 pretreatment of wild type PGECs (Gilman et al., 2019). Prostaglandins modulate inflammatory responses by altering cytokine production and secretion in macrophages (Ricciotti and Fitzgerald, 2011; Aoki and Narumiya, 2012). The signaling pathway downstream of cyclooxygenase-2 (COX-2), the rate-limiting enzyme that converts arachidonic acid into prostaglandins (Chandrasekharan and Simmons, 2004), has been shown to contribute to the IR-induced bystander effect in other cell types (Zhou et al., 2005; Chai et al., 2013; Kobayashi and Konishi, 2018) and P2X7R activation has been shown to induce arachidonic acid release from rat SMG ductal cells (Alzola et al., 1998). These findings suggest that P2X7R antagonists provide radioprotection by attenuating the damaging tissue response to IR-induced release of alarmins, including ATP and PGE_2_.

## P2 Receptors in Salivary Gland Regeneration

While most current treatments for salivary gland dysfunction target expansion of residual salivary acinar cells to repair damaged tissue, regenerative therapy with stem cells is a novel and promising therapeutic approach to replace damaged salivary glands (Carpenter and Cotroneo, 2010; Lombaert et al., 2017; Ogawa and Tsuji, 2017). Several studies have identified and characterized subsets of endogenous salivary progenitor cells that can be exploited to promote tissue regeneration (Lombaert et al., 2008; Chibly et al., 2014, 2018; Pringle et al., 2016; Emmerson et al., 2018; Weng et al., 2018). The use of modified fibrin hydrogels (Nam et al., 2019a), layered sheets of isolated salivary gland cells released from thermoresponsive culture dishes (Nam et al., 2019b) and salivary organoid cultures generated from embryonic pluripotent stem cells (Tanaka et al., 2018) have been explored as regenerative therapies for damaged salivary glands. Tissue engineering of 3-dimensional (3-D) primary HSG cultures for transplantation into afflicted patients represents another regenerative strategy to restore salivary gland function (Lombaert et al., 2017). Because primary human salivary gland cells undergo loss of cell-specific protein expression and biological function when cultured in a monolayer (Jang et al., 2015), development of 3-D culture strategies using Matrigel (Feng et al., 2009; Maria et al., 2011), collagen-Matrigel (Joraku et al., 2007; Pringle et al., 2016), hyaluronic acid-based hydrogels (Pradhan-Bhatt et al., 2013) and magnetic 3-D levitation (Ferreira et al., 2019) has been explored to maintain salivary gland cell function in culture. Indeed, transplantation of 3-D cultured, primary human salivary gland cells has been shown to ameliorate radiation-induced salivary gland dysfunction in mice (Pringle et al., 2016).

Rodent salivary glands have been shown to possess a high capacity to regenerate following the ligation or obstruction of the main excretory ducts of the gland, where ligated salivary glands initially become inflamed before glandular atrophy occurs through TGF-β-induced fibrosis and Fas ligand-induced epithelial cell apoptosis (Burford-Mason et al., 1993; Ahn et al., 2000; Takahashi et al., 2004, 2005, 2007; Carpenter et al., 2007; Woods et al., 2015). Following de-ligation, residual cells in damaged salivary glands can regenerate the gland through proliferation, migration and self-organization (Takahashi et al., 1998; Man et al., 2001; Kishi et al., 2006; Aure et al., 2015), thereby restoring salivary gland function, i.e., increasing the secretion rate of saliva with a normal ion and protein composition (Scott et al., 1999; Osailan et al., 2006). Concurrent with these glandular changes, functional P2Y_2_R expression, which is very low under homeostatic conditions, is robustly increased in salivary epithelial cells in response to ductal ligation and P2Y_2_R expression returns to basal low levels following de-ligation and subsequent recovery of the salivary gland (Ahn et al., 2000; El-Sayed et al., 2014). These findings are in agreement with previous studies demonstrating P2Y_2_R upregulation in epithelial cells in response to tissue damage and inflammation (Turner et al., 1997; Schrader et al., 2005; Degagne et al., 2009; Woods et al., 2018), suggesting that the P2Y_2_R is an important component in the repair and regeneration of damaged salivary glands.

Previous studies have demonstrated a role for the P2Y_2_R in corneal epithelial wound healing by increasing cell migration (Boucher et al., 2010), in liver regeneration by stimulating hepatocyte proliferation (Tackett et al., 2014), in cardiac regeneration by stimulating cardiac progenitor cell proliferation (Khalafalla F.G. et al., 2017) and in intestinal epithelial cell tubulogenesis (Ibuka et al., 2015). Activation of P2Y_2_Rs in the HSG cell line also induces the transactivation, homodimerization and autophosphorylation of the EGFR, a receptor tyrosine kinase known to be crucial for salivary gland branching morphogenesis and development (Miyazaki et al., 2004; Patel et al., 2006; Mizukoshi et al., 2016). This process in salivary epithelial and endothelial cells involves ADAM10/17-dependent proteolytic cleavage induced by P2Y_2_R activation that causes the release of cell surface-bound EGFR ligands as well as the Src kinase-dependent transactivation of growth factor receptors through the binding of Src to SH3 binding motifs in the P2Y_2_R intracellular domain (Liu et al., 2004; Seye et al., 2004; Ratchford et al., 2010). In HSG cells, P2Y_2_R activation also induces the heterodimerization of EGFR and ErbB3, another member of the EGFR family (Ratchford et al., 2010). ErbB3 has an inactive kinase domain that requires heterodimerization with EGFR to respond to its ligand, neuregulin, which then stimulates the ERK/MAPK signaling pathway to promote cell proliferation, migration, and differentiation (Patel et al., 2006; Ratchford et al., 2010).

Integrins are transmembrane cell surface receptors that interact with extracellular matrix components, including laminin (Nishiuchi et al., 2006), fibronectin (Bharadwaj et al., 2017) and collagen (Tuckwell and Humphries, 1996), intracellular cytoskeletal proteins and other cell surface receptors (Legate et al., 2009) that are crucial components in the salivary gland regeneration process (Wei et al., 2007; El-Sayed et al., 2014). Hence, the bi-directional nature of integrin signaling regulates many physiological processes relevant to salivary gland regeneration, including cell proliferation, polarity, migration, and adhesion (Legate et al., 2009). Through its extracellular RGD domain, the P2Y_2_R can bind directly to integrins (e.g., α_v_β_*3/5*_) and allow for nucleotide-induced P2Y_2_R-mediated activation of integrin signaling pathways, including Rho and Rac GTPase activation that regulate cytoskeletal rearrangements (Erb et al., 2001; Wang et al., 2005). The extracellular ligand for the α_5_β_1_ integrin is fibronectin, a well-known mediator of salivary gland morphogenesis (Sakai et al., 2003; Onodera et al., 2010), and we have previously demonstrated that UTP-induced P2Y_2_R activation also induces α_5_β_1_ integrin-mediated migration, aggregation, and self-organization of dispersed salivary epithelial cells into acinar-like spheres (El-Sayed et al., 2014). These spheres resemble native acinar units of the salivary gland, possessing a lumen and organized expression of the tight junction protein ZO-1, and we have shown that the mechanism for P2Y_2_R-mediated self-organization of salivary gland cells involves the activation of EGFR via the Cdc42 Rho GTPase pathway and subsequent downstream activation of ERK1/2 and JNK signaling pathways (El-Sayed et al., 2014). Thus, these studies suggest a promising role for unique structural motifs in P2Y_2_Rs that are highly relevant to cell-based regenerative therapy and bioengineering of salivary glands.

## Summary

Activation of purinergic receptors for extracellular nucleotides in the salivary glands modulates various physiological and pathophysiological functions (Table 1). The ATP-gated ionotropic P2X7 receptor in salivary acinar cells contributes to physiological salivary gland function by modulating muscarinic receptor-induced saliva secretion into the ductal lumen, whereas activation of ductal P2X7Rs modulates ion and protein content of saliva. P2X4R activation also contributes to saliva secretion through the formation of functional homotrimers and P2X4R/P2X7R heterotrimers in salivary gland epithelium, suggesting that P2XRs represent an integration point between canonical and non-canonical signaling pathways that regulate saliva flow and composition. P2Y_1_Rs also may contribute to salivary gland development through coupling to multiple G proteins resulting in diverse physiological responses. The ability of P2Y_2_R activation to stimulate increases in [Ca^2+^]_i_ and Cl^–^ flow across epithelial membranes suggests a role in saliva secretion, however, P2Y_2_R expression is negligible under normal steady-state conditions. The observed upregulation of P2Y_2_R expression during tissue stress and in response to P2X7R-induced IL-1β release suggest their significant role in salivary gland pathophysiology. Due to an increase in extracellular nucleotide release during tissue inflammation and dysregulation, nucleotide-induced activation of the interconnected P2X7R-P2Y_2_R signaling pathways likely modulates multiple immunological and tissue repair functions, including cell migration, growth factor receptor transactivation, integrin signaling, adhesion molecule upregulation, and cytokine release. Thus, P2X7R activation in salivary epithelium and upregulation of the P2Y_2_R with its unique structural domains likely regulate both salivary gland dysfunction and repair through the stimulation of these important pro-inflammatory processes.

**TABLE 1:** Expression and function of purinergic receptors in salivary glands.

Purinergic receptor	Cell or tissue type	Salivary gland function	References
P2X7	Rat parotid acinar cells	Mediates eATP-induced Ca^2+^ entry	Soltoff et al., 1990; Dehaye, 1993; McMillian et al., 1993, 1987
		Mediates eATP-induced plasma membrane permeabilization and large pore formation	Gibbons et al., 2001
	Rat submandibular acinar cells	Induces plasma membrane permeabilization and large pore formation	Alzola et al., 2001
		Inhibits carbachol- and substance P-induced mobilization of intracellular Ca^2+^	Hurley et al., 1993; Metioui et al., 1996
		Increases phospholipase A2-dependent secretion of arachidonic acid and kallikrein	Alzola et al., 1998
	Rat submandibular acinar and ductal cells	Mediates eATP-induced Ca^2+^ entry and increases membrane Cl^–^ conductance	Lee et al., 1997
	Mouse parotid acinar cells	Modulates carbachol-induced Ca^2+^ mobilization	Novak et al., 2010
		Mediates eATP-induced Ca^2+^ entry, Ca^2+^-induced Ca^2+^ release, and exocytosis	Bhattacharya et al., 2015, 2012
		Mediates eATP-induced membrane anion conductance	Reyes et al., 2008
	Mouse parotid acinar and ductal cells	Mediates eATP-induced Ca^2+^ entry and membrane conductance; cell-specific channel assembly properties	Li et al., 2003
		Mediates γ-radiation induced eATP and PGE_2_ release	Gilman et al., 2019
	Mouse submandibular acinar and ductal cells	Mediates eATP-induced apoptosis, ROS production, NLRP3 inflammasome assembly and IL-1β release	Woods et al., 2012; Khalafalla M.G. et al., 2017
	*Ex vivo* mouse submandibular gland	Mediates eATP-induced fluid secretion and inhibits carbachol-induced fluid secretion	Nakamoto et al., 2009
	*In vivo* mouse salivary glands	Modulates carbachol-induced saliva secretion	Pochet et al., 2007; Novak et al., 2010

P2X4	Rat parotid acinar cells	Mediates eATP-induced Ca^2+^ entry	McMillian et al., 1993
	Mouse parotid acinar cells	Mediates eATP-induced Ca^2+^ entry and exocytosis; potentiated by increased cAMP levels	Bhattacharya et al. 2012; 2015
		Mediates eATP-activated membrane currents; functional interaction with P2X7 receptor	Casas-Pruneda et al., 2009
	Mouse submandibular ductal cells	Mediates eATP-induced Ca^2+^ entry	Pochet et al., 2007
	Human parotid acinar cells	Mediates eATP-induced Ca^2+^ entry; potentiated by increased cAMP levels	Brown et al., 2004

P2Y_1_	Rat submandibular acinar and ductal cells	Mediates nucleotide-induced [Ca^2+^]_i_ increase; decreased activity in aged animals	Park et al., 1997
		Mediates nucleotide-induced [Ca^2+^]_i_ increase and ERK1/2 phosphorylation; differential coupling to Gα_14_ and Gα_q/11_ during development	Baker et al., 2006

P2Y_2_	Rat parotid cell line ParC10	Mediates eUTP-induced increase in short-circuit current and Cl^–^ efflux	Turner et al., 1998a
	Rat submandibular acinar and ductal cells	Mediates eUTP-induced increase in membrane Cl^–^ conductance	Lee et al., 1997; Zeng et al., 1997
		Increased expression and eUTP-induced [Ca^2+^]_i_ increase during short-term culture	Turner et al., 1997
	*In vivo* rat submandibular glands	Increases CFTR-mediated Cl^–^ reabsorption to modify saliva ion content	Ishibashi et al., 2008
	Mouse submandibular acinar and ductal cells	Mediates eUTP-induced cell aggregation and migration through EGFR transactivation	El-Sayed et al., 2014
	*In vivo* mouse submandibular glands	Increased expression and eUTP-induced [Ca^2+^]_i_ increase during salivary gland inflammation	Schrader et al., 2005; Ahn et al., 2000; Woods et al., 2018
	Human salivary gland (HSG) cell line	Mediates UTP-induced IP_3_ production, [Ca^2+^]_i_ increase and K^+^ efflux	Yu and Turner, 1991
		Potentiates cell regulatory volume decrease in response to hypotonic stress	Kim et al., 1996
		Increases vascular cell adhesion molecule expression	Baker et al., 2008
		Mediates eUTP-induced EGFR phosphorylation and induces EGFR and ErbB3 heterodimerization	Ratchford et al., 2010

*eATP, extracellular ATP; eUTP, extracellular UTP; PGE_2_, prostaglandin E_2_; CFTR, cystic fibrosis transmembrane conductance regulator; EGFR, epidermal growth factor receptor.*

In conclusion, purinergic receptors have emerged as promising therapeutic targets to promote physiological saliva flow, prevent salivary gland inflammation and enhance tissue regeneration required to reverse common causes of salivary gland dysfunction in humans, such as the autoimmune disease SS or the side effect of radiotherapy in head and neck cancer patients. Because purinergic receptors share common agonists and form heteromeric receptors with distinct pharmacologic profiles, unraveling the contribution of intracellular P2 receptor cross-talk to salivary gland dysfunction in animal models and humans will further define their therapeutic value in the treatment of salivary gland disorders. The continued development of high affinity P2R agonists and antagonists and the investigation of their safety and efficacy represent the next steps in the clinical translation of this promising P2 receptor research.

## Author Contributions

MK, LW, KJ, KF, and JC reviewed literature and drafted the manuscript. MK, LW, KJ, KF, JC, JJ, KL, HG, and GW critically revised, edited, and approved the manuscript.

## Conflict of Interest

The authors declare that the research was conducted in the absence of any commercial or financial relationships that could be construed as a potential conflict of interest.
